# Ultrasound-Derived Abdominal Muscle Thickness Better Detects Metabolic Syndrome Risk in Obese Patients than Skeletal Muscle Index Measured by Dual-Energy X-Ray Absorptiometry

**DOI:** 10.1371/journal.pone.0143858

**Published:** 2015-12-23

**Authors:** Ayumi Ido, Yuki Nakayama, Kojiro Ishii, Motoyuki Iemitsu, Koji Sato, Masahiro Fujimoto, Toshiyuki Kurihara, Takafumi Hamaoka, Noriko Satoh-Asahara, Kiyoshi Sanada

**Affiliations:** 1 College of Sport and Health Science, Ritsumeikan University, Shiga, Japan; 2 Faculty of Health and Sports Science, Doshisha University, Kyoto, Japan; 3 Department of Sports Medicine for Health Promotion, Tokyo Medical University, Tokyo Japan; 4 Division of Diabetic Research, Clinical Research Institute, National Hospital Organization Kyoto Medical Center, Kyoto, Japan; INIA, SPAIN

## Abstract

Sarcopenia has never been diagnosed based on site-specific muscle loss, and little is known about the relationship between site-specific muscle loss and metabolic syndrome (MetS) risk factors. To this end, this cross-sectional study aimed to investigate the relationship between site-specific muscle size and MetS risk factors. Subjects were 38 obese men and women aged 40–82 years. Total body fat and lean body mass were assessed by whole-body dual-energy X-ray absorptiometry (DXA) scan. Muscle thickness (MTH) was measured using B-mode ultrasound scanning in six body regions. Subjects were classified into general obesity (GO) and sarcopenic obesity (SO) groups using the threshold values of one standard deviation below the sex-specific means of either MTH or skeletal muscle index (SMI) measured by DXA. MetS risk score was acquired by standardizing and summing the following continuously distributed variables: visceral fat area, mean blood pressure, HbA1c, and serum triglyceride / high density lipoprotein cholesterol, to obtain the Z-score. Multiple regression analysis revealed that the MetS risk score was independently associated with abdominal MTH in all subjects, but not with MTH in other muscle regions, including the thigh. Although HbA1c and the number of MetS risk factors in the SO group were significantly higher than those in the GO group, there were no significant differences between GO and SO groups as defined by SMI. Ultrasound-derived abdominal MTH would allow a better assessment of sarcopenia in obese patients and can be used as an alternative to the conventionally-used SMI measured by DXA.

## Introduction

Sarcopenia, which refers to the decline in muscle mass and strength with age, leads to several disabilities [1–3] and lifestyle-related diseases [4–7]. The coexistence of sarcopenia and obesity is referred to as sarcopenic obesity (SO). This condition is associated with a higher risk for metabolic syndrome (MetS) than obesity or sarcopenia alone [8,9].

Although muscle mass may decline by 25% between the ages of 50 and 75 years [10], the extent of age-related loss of muscle mass appears to vary across body regions. Previous studies have demonstrated that greater rates of age-related loss of skeletal muscle (SM) mass occur in the thigh and abdominal regions, whereas only moderate losses occur in the upper-trunk and arm regions [11–13]. In particular, age-related thigh muscle volume loss is muscle-specific, in that a greater degree of quadriceps muscle loss is evident in older individuals [14]. These findings suggest that assessing site-specific muscle loss in the thigh and abdominal regions would better detect obese individuals at higher MetS risk than assessing total body muscle loss.

Diagnostic imaging techniques including computed tomography (CT), magnetic resonance imaging (MRI), and ultrasound scanning have been widely used to directly and accurately assess muscle size [15–21]. Of these techniques, ultrasound-derived muscle thickness (MTH) can be used to accurately predict total and/or regional SM mass [22]. Since ultrasound scanning is cheaper, more portable, and more widely available than the other techniques, it holds the potential for widespread use as a clinical screening tool to identify individuals with sarcopenia.

At present, however, sarcopenia has never been diagnosed by site-specific muscle loss, and little is known about the relationship between site-specific muscle loss and MetS risk factors. To this end, this study aimed to investigate the relationship between site-specific muscle size and MetS risk factors. We hypothesized that muscle size in the thigh and abdominal regions, compared to other regions, would better detect MetS risk in obese patients.

## Materials and Methods

### Subjects

Subjects were Japanese men (n = 16) and women (n = 22) aged 40–82 years who visited the National Hospital Organization Kyoto Medical Center. All subjects had a body mass index (BMI) >25 kg/m^2^, were medically examined by a physician, and were excluded if they were pregnant or had cardiovascular disease, cerebrovascular disease, severe mental disorder, or a physical disability.

The purpose, procedures, and risks of the study were explained to each subject and all subjects provided written informed consent prior to participation. The study was approved by the Institutional Review Board of Ritsumeikan University (BKC-IRB-2013-055).

### Analysis of blood samples

All blood samples were collected from subjects in the seated position. Fasting (>12 h) blood samples were collected by venipuncture in tubes with or without ethylenediaminetetraacetic acid, refrigerated immediately, and centrifuged at 1500 rpm for 30 min at 4°C within 2 h. Samples were stored at –20°C until use. Serum concentrations of total cholesterol and triglyceride were determined using commercial kits (Mitsubishi Chemical Medience, Tokyo, Japan). Serum high-density lipoprotein (HDL) cholesterol was measured by an enzymatic method (Mitsubishi Chemical Medience). Serum low-density lipoprotein (LDL) cholesterol was calculated as follows: total cholesterol (mg/dl)–HDL cholesterol (mg/dl)–triglyceride (mg/dl) × 0.2 [23]. Plasma glucose was measured using the glucose dehydrogenase method [24]. Whole-blood glycohemoglobin A1c (HbA1c) was measured by an enzymatic method (Glycohemoglobin A1c kit; Mitsubishi Chemical Medience).

### Analysis of arterial blood pressure at rest

Systolic blood pressure, mean blood pressure, and diastolic blood pressure were measured at rest using a vascular testing device (Form PWV/ABI; Colin Medical Technology, Komaki, Japan). Chronic arterial blood pressure levels at rest were measured with the same device over the brachial and dorsalis pedis arteries. Recordings were made in triplicate with subjects in the supine position. Brachial-ankle pulse wave velocity (baPWV), which provides qualitatively similar information to that derived from central arterial stiffness [25], was measured by the volume plethysmographic method.

### Metabolic syndrome (MetS) risk

In addition to the accumulation of visceral fat (waist circumference ≥ 85 cm in men, ≥ 90 cm in women), subjects with two or more of the following risk factors were considered to have metabolic syndrome: high systolic and diastolic blood pressure (≥ 130 mmHg for systolic blood pressure and/or ≥ 85 mmHg for diastolic blood pressure), abnormal serum lipids (≥ 150 mg/dl for triglyceride and/or < 40 mg/dl for HDL cholesterol), and high plasma glucose (≥ 110 mg/dl). The MetS risk score was derived by standardizing and then summing the following continuously distributed variables: visceral fat area, mean blood pressure, HbA1c, and serum triglyceride/HDL cholesterol, to obtain the Z-score.

### Whole-body dual-energy X-ray absorptiometry

Total body fat and lean body mass were assessed using dual-energy X-ray absorptiometry (DXA; Lunar Prodigy, GE Healthcare, Tokyo, Japan). Participants were positioned for whole-body scans in accordance with the manufacturer’s protocol. Participants lay in a supine position on the DXA table with limbs close to the body. The whole-body lean soft tissue mass was divided into several regions, i.e., arms, legs, and the trunk. Appendicular muscle mass (AMM) was estimated as the sum of lean soft tissue of the two upper limbs and two lower limbs. Reference values [skeletal muscle index, SMI; AMM/height^2^, kg/m^2^] for sarcopenia for each sex were defined as values 1 SD below the sex-specific means of study reference data for young adults aged 18–40 years, respectively.

### MRI

Visceral adipose tissue (VAT) and subcutaneous adipose tissue (SAT) were determined using an axial spin-echo T1-weighted MRI system (Signa HDxt 1.5T, GE Healthcare, USA). Images were taken using an 8-channel body array coil with the following parameters: echo time/repetition time = 7 ms/respiration; matrix = 384 × 384; field of view = 420 × 420 mm; slice thickness = 10 mm; gap = 0 mm; number of excitations = 2. A single-slice MRI through the abdomen at the level between L4 and L5 was obtained, and VAT and SAT areas were determined using image analysis software (SliceOmatic Ver.4.3, Tomovision Inc., Montreal, Canada).

### Ultrasound

B-mode ultrasound MTHs were taken at six sites (anterior and posterior upper arm, abdomen, subscapula, and anterior and posterior thigh) from the anterior and posterior surfaces of the body as described previously by Sanada et al. [22]. Six anatomical landmarks for the sites are noted below.

#### Anterior and posterior upper arm

On the anterior and posterior surface, 60% distal between the lateral epicondyle of the humerus and the acromial process of the scapula.

#### Abdomen

At a distance 2–3 cm to the right of the umbilicus.

#### Subscapula

At a distance of 5 cm, directly below the inferior angle of the scapula.

#### Anterior and posterior thigh

On the anterior and posterior surface, midway between the lateral condyle of the femur and the greater trochanter.

**Fig 1:**
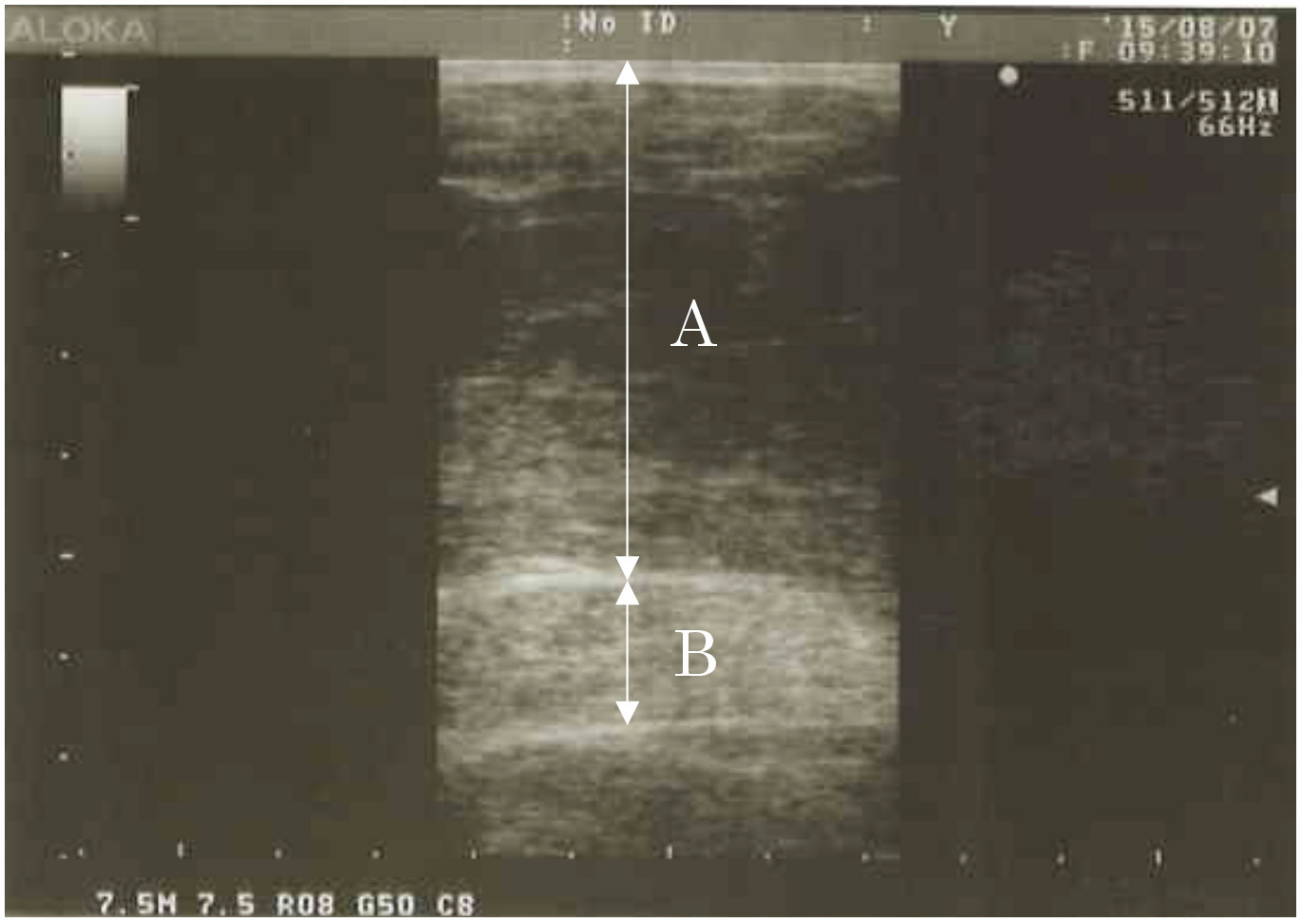
Typical abdominal ultrasound image in obesity patient. The thickness values of the muscle tissues was determined as the distances between the skin and fat—muscle tissue interface and between the fat—muscle tissue interface and muscle—abdominal cavity boundary. (A) Subcutaneous fat thickness, (B) Muscle thickness.

MTHs were scanned using a real-time linear electronic probe of 7.5 MHz (SSD-500, Aloka, Tokyo, Japan). The scanning head was prepared with water-soluble transmission gel that provided acoustic contact without depression of the skin surface. The scanner was placed perpendicular to the tissue interface at the marked sites. MTH was measured directly from the screen using electronic calipers and determined to be the distance from the adipose tissue—muscle interface to the muscle—bone interface. The reliability of image reconstruction and distance measurements was confirmed by comparing the ultrasonic and manual measurements of tissue thickness in human cadavers, and the CV of this MTH measurement was 1.0% [26]. Ultrasound muscle thickness in obesity patients is not established, however, in Japanese sumo wrestler (BMI = 38.2 and %fat = 33.9), the CV of this method from test-retest (20 samples from sumo wrestler) was 1.2% [27]. Typical abdominal ultrasound image in obesity patient showed in Fig 1. The thickness values of the muscle tissues was determined as the distances between the skin and fat—muscle tissue interface and between the fat—muscle tissue interface and muscle—abdominal cavity boundary. These border lines is clearly confirmable in obesity patients as is the case in healthy subjects.

Cut-off values of the six sites of sarcopenia by ultrasound were defined as described in a previous study [28], which assessed 413 Japanese men (n = 182) and women (n = 231) aged 20–40 years. The cut-off values for class 1 sarcopenia (1 SD below sex-specific means) in Japanese men and women are shown in Table 1. All subjects were classified into general obesity (GO) and SO groups based on a cut-off value defined by abdominal MTH.

**Table 1:** Reference values for sarcopenia using ultrasound MTH and SMI.

Male	Anterior upper arm MTH^1^ (mm)	Posterior upper arm MTH^1^ (mm)	Subscapular MTH^1^ (mm)	Abdominal MTH^1^ (mm)	Anterior thigh MTH^1^ (mm)	Posterior thigh MTH^1^ (mm)	SMI^2^ (kg/m^2^)
Mean	31.8	36.2	27.3	13.8	53.3	64.9	8.67
SD	3.4	5.9	6.5	2.7	7.6	7.9	0.90
Reference value	28.4	30.4	20.8	11.1	45.7	57.1	7.77
Female	Anterior upper arm MTH^1^ (mm)	Posterior upper arm MTH^1^ (mm)	Subscapular MTH^1^ (mm)	Abdominal MTH^1^ (mm)	Anterior thigh MTH^1^ (mm)	Posterior thigh MTH^1^ (mm)	SMI^2^ (kg/m^2^)
Mean	22.1	23.6	18.2	10.1	44.2	52.8	6.78
SD	3.2	4.6	4.3	1.8	5.8	5.6	0.66
Reference value	18.9	18.9	13.9	8.3	38.4	47.2	6.12

MTH, Muscle thickness; SMI, Skeletal muscle index. Data were obtained from:

^1^Sanada et al. (2007, Reference #27),

^2^Sanada et al. (2010, Reference #36).

### Handgrip strength

Handgrip strength was measured using a handheld dynamometer (GRIP-D, Takei Scientific Instruments Co., Niigata, Japan). In the standing position, with the arms straight by the sides, subjects gripped the dynamometer as hard as possible for 3 sec. without pressing the instrument against the body or bending at the elbow. Two trials were performed with each hand. The average of the higher value with each hand was recorded.

### Statistical analysis

All measurements and calculated values are expressed as mean ± SD or median [interquartile range (IQR)]. Student’s unpaired *t* test was performed to test gender differences. Some of the data were not normally distributed; therefore, in these cases, the nonparametric Mann-Whitney U test was used instead of the t-test. The Shapiro-wilk test was used to verify whether the distribution of variables followed a Gaussian pattern. Multiple regression analysis was performed with MetS risk score as a dependent variable and MTHs as independent variables. Partial correlation coefficients between body weight and site-specific muscle thickness adjusted for sex and age were calculated. Mean values for general criteria, body composition values, physical characteristics, blood measurements, arteriosclerosis, MetS-related parameters, and functional performance were compared between GO and SO groups by one-way ANCOVA, with adjustments for covariates of sex, age, and BMI. The alpha level for testing significance was set at *P* < 0.05. All statistical analyses were performed using StatView ver.5.0 for Windows (SAS Institute, NC, USA).

## Results

The physical characteristics of subjects are summarized in Table 2. There were no significant gender differences in age and BMI. MetS-related parameters and functional performance are summarized in Table 3. There were no significant gender differences in HbA1c and MetS risk score. Multiple regression analysis revealed that MetS risk score was independently associated with abdominal MTH in all subjects (β = -0.607, *P* < 0.01), but not with MTHs in the other muscle regions, including the thigh (Table 4).

**Table 2:** Physical characteristics of subjects.

	All (n = 38)	Female (n = 22)	Male (n = 16)
Age (years)	58.3 ± 11.8	56.9 ± 10.3	60.4 ± 13.7
Height (cm)	162.4 ± 8.3	157.5 ± 5.2	169.3 ± 6.7***
Weight (kg)	74.2 [67.7–89.4]	72.2 [64.6–89.8]	83.5 [72.1–91.4]
BMI (kg/m^2^)	29.5 [26.2–31.3]	30.3 [26.3–35.1]	28.3 [26.0–30.5]
Whole body fat percentage (%)	37.3 ± 7.3	42.1 ± 4.3	30.7 ± 5.0***
Waist circumference (cm)	100.8 ± 11.6	102.7 ± 13.0	98.3 ± 9.1
AMM (kg)	18.6 [16.0–24.2]	16.6 [15.5–18.1]	23.9 [20.9–26.5]***
SMI (kg/m^2^)	7.5 ± 1.1	7.0 ± 1.0	8.3 ± 0.8***
VFA (cm^2^)	133.7 ± 53.2	127.8 ± 55.1	141.7 ± 51.0
SFA (cm^2^)	318.7 ± 126.3	370.1 ± 129.5	248.0 ± 81.7**
Anterior upper arm MTH (mm)	27.7 ± 4.3	26.1 ± 4.2	29.9 ± 3.4**
Posterior upper arm MTH (mm)	29.8 ± 5.1	29.5 ± 5.7	30.3 ± 4.4
Subscapular MTH (mm)	25.9 ± 5.9	25.4 ± 6.7	26.5 ± 4.7*
Abdominal MTH (mm)	10.1 ± 2.3	9.5 ± 2.1	11.1 ± 2.2**
Anterior thigh MTH (mm)	49.2 ± 8.2	48.6 ± 6.8	50.1 ± 10.0
Posterior thigh MTH (mm)	55.9 ± 8.4	57.2 ± 8.1	54.1 ± 8.6

Data are means±SD or median [IQR]. MTH, Muscle thickness; BMI, Body mass index; AMM, Appendicular muscle mass; SMI, Skeletal muscle index; VFA, Visceral fat area; SFA, Subcutaneous fat area.

* *P* < 0.05,

** *P* < 0.01,

*** *P* < 0.001 for the significant difference from female using unpaired Student's t-test or Mann-whitny U test.

**Table 3:** MetS-related parameter and functional measurement.

	All (n = 38)	Female (n = 22)	Male (n = 16)
SBP (mmHg)	132.7 ± 12.9	136.3 ± 12.4	127.8 ± 12.4*
DBP (mmHg)	80.9 ± 9.1	81.5 ± 9.1	80.1 ± 9.4
MBP (mmHg)	101.7 ± 10.5	103.4 ± 10.1	99.3 ± 11.0
Fasting plasma glucose (mg/dL)	104 [94–129]	103 [97–115]	114 [93–139]
HbA1c (%)	6.1 [5.7–6.5]	6.1 [5.7–6.4]	6.0 [5.4–7.4]
Triglyceride (mg/dL)	113 [76–155]	93 [72–134]	150 [109–213]*
Total cholesterol (mg/dL)	185 [172–209]	184 [173–203]	198 [171–235]
HDL-cholesterol (mg/dL)	55.1 ± 10.8	58.7 ± 10.9	50.3 ± 8.8*
LDL-cholesterol (mg/dL)	115.2 ± 30.9	113.9 ± 24.8	117.1 ± 38.6
baPWV (cm/s)	1469.6 ± 249.2	1450.6 ± 255.0	1495.7 ± 246.8
Triglyceride / HDL-cholesterol	2.1 [1.2–3.5]	1.6 [1.2–2.7]	2.9 [1.8–5.3]*
MetS risk, Z-score	0.0 ± 2.5	0.0 ± 2.6	0.0 ± 2.6
Number of MetS risk factors (n)	2.9 ± 0.9	2.7 ± 0.8	3.1 ± 1.0
Hand grip strength (kg)	27.3 [23.9–34.2]	24.4 [22.4–27.8]	36.3 [28.4–44.6]***

Data are means±SD or median [IQR]. MetS, Metabolic syndrome; SBP, Systolic blood pressure; DBP, Diastolic blood pressure; MBP, Mean blood pressure; HDL, High density lipoprotein; LDL, Low density lipoprotein; baPWV, brachial-ankle pulse wave velocity.

* *P* < 0.05,

*** *P* < 0.001 for the significant difference from female using unpaired Student's *t*-test or Mann-whitny U test.

**Table 4:** Relationship of MetS risk score to variable using multiple regression analysis.

Variables (mm)	Standardized regression coefficient(β)
Anterior upper arm MTH	0.178
Posterior upper arm MTH	0.058
Subscapular MTH	0.199
Abdominal MTH	–0.607**
Anterior thigh MTH	–0.381
Posterior thigh MTH	0.361

MetS, Metabolic syndrome; MTH, Muscle thickness.

** *P* < 0.01.

We observed significant positive partial correlations between body weight and anterior upper arm MTH (r = 0.333, *P* < 0.05), posterior upper arm MTH (r = 0.401, *P* < 0.01), anterior thigh MTH (r = 0.597, *P* < 0.001), posterior thigh MTH (r = 0.572, *P* < 0.001), and SMI (r = 0.604, *P* < 0.001), after adjusting for sex and age. No significant partial correlations were found between body weight and MTH for subscapular (r = 0.249, *P* = 0.143) and abdominal regions (r = 0.176, *P* = 0.305).

**Fig 2:**
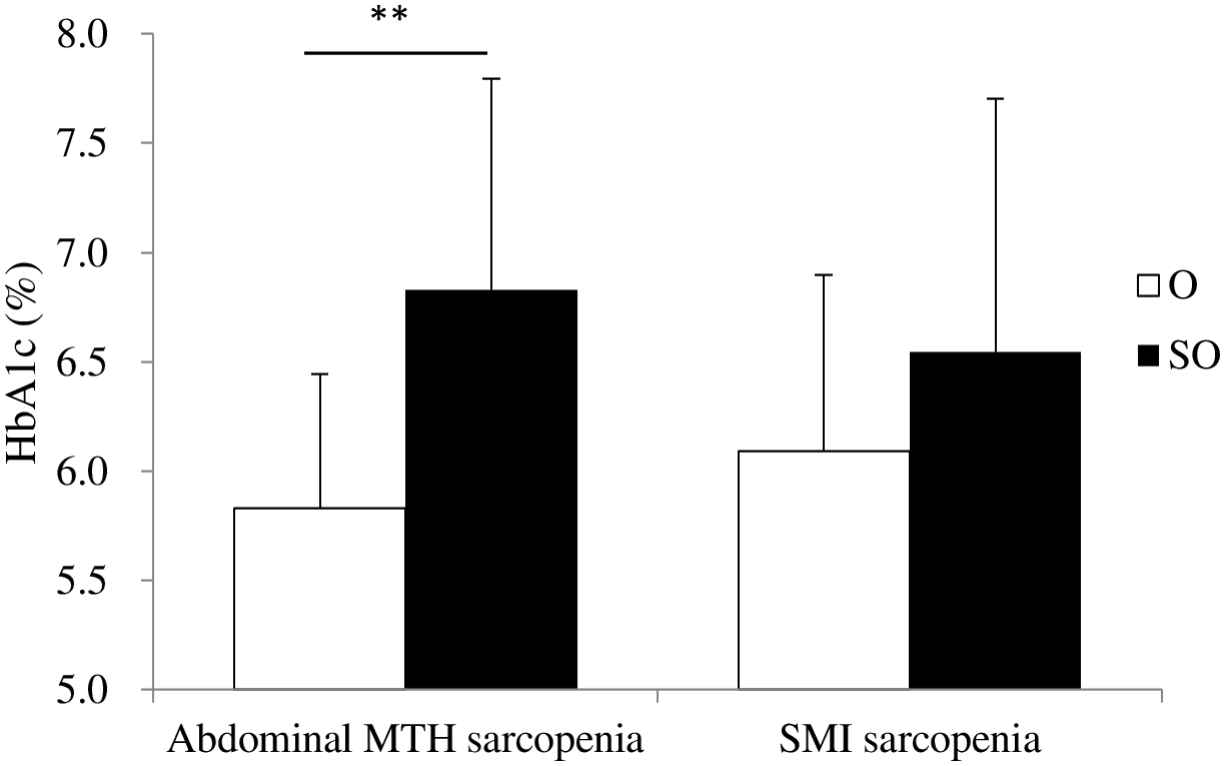
Comparison of HbA1c between GO and SO groups, as defined by abdominal MTH and SMI. GO, General obesity; SO, Sarcopenic obesity; MTH, Muscle thickness; SMI, Skeletal muscle index. ** *P* < 0.01 for significant differences relative to the GO group using ANCOVA, with adjustments for covariates of sex, age, and body mass index.

**Fig 3:**
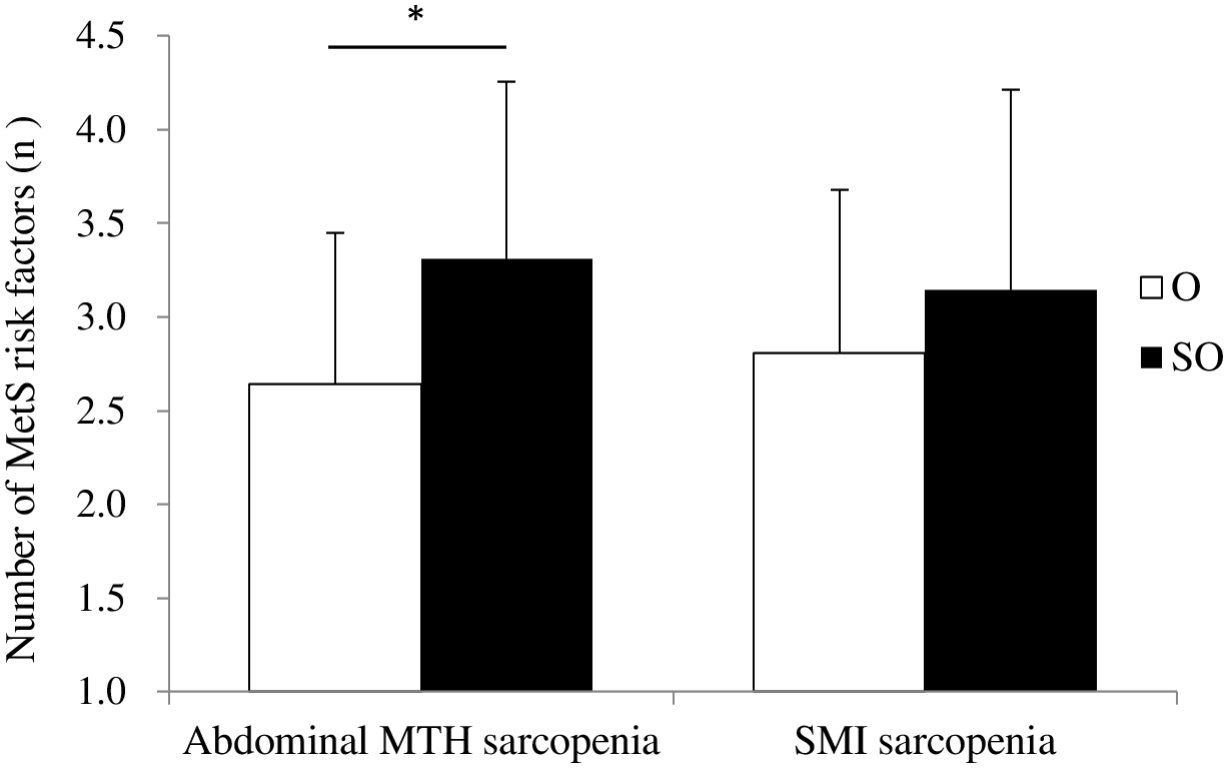
Comparison of the number of MetS risk factors between GO and SO groups, as defined by abdominal MTH and SMI. GO, General obesity; SO, Sarcopenic obesity; MetS, Metabolic syndrome; MTH, Muscle thickness; SMI, Skeletal muscle index. * *P* < 0.05 for significant differences relative to the GO group using ANCOVA, with adjustments for covariates of sex, age, and body mass index.

Table 5 shows MetS-related parameters and functional performance between GO and SO groups, as defined by abdominal MTH and SMI. Although HbA1c in the SO group was significantly higher than that in the GO group (*P* < 0.001), there was no significant difference in HbA1c between the two groups, as defined by DXA-measured SMI (Fig 2). Similarly, although the number of MetS risk factors in the SO group, as defined by abdominal MTH, was significantly higher than that in the GO group (*P* < 0.05), there was no significant difference in the number of MetS risk factors between GO and SO groups, as defined by DXA-measured SMI (Fig 3). In addition, handgrip strength in the SO group, as defined by abdominal MTH, was significantly lower than that in the GO group (*P* < 0.05). However, there was no significant difference in handgrip strength between GO and SO groups, as defined by DXA-measured SMI. Conversely, baPWV in the SO group, as defined by SMI, was significantly lower than that in the GO group (*P* < 0.05). However, no significant difference in baPWV was found between GO and SO groups, as defined by abdominal MTH.

**Table 5:** Comparison of physical characteristics and MetS-related parameter between GO and SO defined by abdominal MTH and SMI.

	Abdominal MTH sarcopenia		SMI sarcopenia	
	GO	SO	GO	SO
n (Male/Female)	25 (8/17)	13 (8/5)	31 (12/19)	7 (4/3)
Age (years)	55.4 ± 11.4	63.9 ± 10.9	57.2 ± 11.3	63.3 ± 13.8
Height (cm)	162.7 ± 9.3	162.0 ± 6.1	162.3 ± 8.3	163.0 ± 8.6
Weight (kg)	80.5 ± 15.1	77.2 ± 12.8	81.4 ± 14.6	70.3 ± 8.5
BMI (kg/m^2^)	30.4 ± 5.1	29.4 ± 4.1	30.8 ± 4.8	26.4 ± 1.6
Whole body fat percentage (%)	38.3 ± 7.3	35.4 ± 7.3	37.7 ± 7.8	35.4 ± 4.6
Waist circumference (cm)	100.9 ± 11.9	100.7 ± 11.4	102.7 ± 11.9	92.7 ± 4.8
VFA (cm^2^)	122.6 ± 50.3	154.9 ± 54.0	131.5 ± 57.2	143.4 ± 30.0
SFA (cm^2^)	341.2 ± 129.9	275.4 ± 111.1	340.4 ± 128.1	222.4 ± 55.8
SBP (mmHg)	132.7 ± 12.9	132.7 ± 13.5	133.0 ± 13.6	131.6 ± 10.2
DBP (mmHg)	81.0 ± 9.5	80.6 ± 8.8	80.9 ± 9.6	80.8 ± 7.3
MBP (mmHg)	101.9 ± 10.7	101.3 ± 10.6	101.5 ± 11.0	102.7 ± 8.8
Fasting plasma glucose (mg/dL)	107.8 ± 21.0	132.2 ± 42.9*	111.0 ± 22.4	138.9 ± 55.1
HbA1c (%)	5.8 ± 0.6	6.8 ± 1.0***	6.1 ± 0.8	6.5 ± 1.2
Triglyceride (mg/dL)	110.6 ± 52.0	165.2 ± 82.9*	118.6 ± 58.5	176.7 ± 92.1*
Total cholesterol (mg/dL)	194.1 ± 30.1	200.4 ± 52.2	193.7 ± 30.4	207.6 ± 66.0
HDL-cholesterol (mg/dL)	55.8 ± 9.3	53.8 ± 13.6	56.2 ± 10.7	50.4 ± 11.1
LDL-cholesterol (mg/dL)	116.2 ± 25.4	113.5 ± 40.7	113.8 ± 24.6	121.8 ± 53.1
baPWV (cm/s)	1415.4 ± 248.2	1573.7 ± 224.4	1416.1 ± 223.6	1706.2 ± 229.6*
Triglyceride / HDL-cholesterol	2.1 ± 1.3	3.4 ± 2.2	2.3 ± 1.4	3.9 ± 2.4*
MetS risk, Z-score	-0.7 ± 2.0	1.4 ± 2.9*	-0.3 ± 2.3	1.5 ± 3.0*
Number of MetS risk factors (n)	2.6 ± 0.8	3.3 ± 0.9*	2.8 ± 0.9	3.1 ± 1.1
Hand grip strength (kg)	30.6 ± 9.8	29.0 ± 6.5*	30.4 ± 9.3	28.1 ± 5.8

Data are means±SD. GO, General obesity; SO, Sarcopenic obesity; MTH, Muscle thickness; BMI, Body mass index; VFA, Visceral fat area; SFA, Subcutaneous fat area, SBP, Systolic blood pressure; DBP, Diastolic blood pressure; MBP, Mean blood pressure; HDL, High density lipoprotein; LDL, Low density lipoprotein; baPWV, brachial-ankle pulse wave velocity; MetS, Metabolic syndrome. Difference between GO and SO were determined by one-way ANCOVA with adjustment for the covariate of sex, age and body mass index.

* *P* < 0.05,

*** *P* < 0.001 v.s. GO.

## Discussion

This study aimed to investigate the relationship between site-specific muscle size and MetS risk factors. We hypothesized that muscle size in the thigh and abdominal regions, compared to other regions, would better indicate MetS risk in obese patients. We found that the MetS risk score was independently associated with abdominal MTH measured by ultrasound, but not with MTH in the other muscle regions, including the thigh. Furthermore, HbA1c and the number of MetS risk factors in the SO group, as defined by abdominal MTH, were significantly higher than that in the GO group, although no significant difference was detected when SO was defined by DXA-measured SMI. These findings suggest that abdominal MTH measured by ultrasound scanning could better detect obese patients at higher MetS risk than by using whole-body skeletal muscle mass.

Although we hypothesized that both anterior thigh and abdominal MTH would be significantly associated with MetS risk, abdominal MTH was the only independent predictor of MetS risk score (Table 4). This could be due to the effect of body weight on lower limbs. Muscle mass in the lower limbs increases as body weight increases to support more weight, whereas muscle mass in the abdominal region appears to be less affected by body weight. In fact, a previous study reported that obese women (BMI >29) had significantly more AMM and leg muscle mass compared to normal-weight subjects (BMI = 24–29) and lean women (BMI <24) [29]. This was corroborated by our findings that abdominal MTH had no significant partial correlation with body weight after adjusting for sex and age, while significant partial correlations were found between body weight and thigh MTH. Thus, although age-related muscle loss in the thigh region is apparent in older adults [14], excess weight in the obese patients of this study may have helped maintain thigh muscle mass, thereby reducing the sensitivity for detecting MetS risk. These results suggest that abdominal muscle thickness measured by ultrasound scanning could be used to detect higher MetS risk in obese patients.

AMM measured by DXA is commonly used to diagnose sarcopenia [30]. However, several studies have shown that the DXA method significantly overestimates muscle mass and underestimates adipose tissue compared to when golden standard imaging techniques are used, i.e., MRI and CT [31–33]. This could be due to the fact that DXA detects intramuscular adipose tissue (IMAT) as lean tissue. IMAT of the thigh muscle, which comprises the majority of AMM, was reported to be positively correlated with BMI [34]. This implies that AMM obtained by DXA would be overestimated due to the increased IMAT measured as lean tissue in obese patients. It has also been reported that IMAT accumulation is muscle specific. For instance, thigh IMAT reportedly increased as BMI increased in some studies [34,35], whereas IMAT at L3, including the rectus abdominis, was not associated with BMI [36]. Thus, abdominal MTH appears to be more suitable for assessing sarcopenia in obese patients, as it excludes the influence of increasing IMAT with obesity. These results suggest that ultrasound-derived abdominal MTH would allow a better assessment of sarcopenia in obese patients and can be used as an alternative to conventionally-used SMI measured by DXA.

In this study, baPWV in the SO group, as defined by SMI, was significantly lower than that in the GO group, while no significant difference was observed between GO and SO groups when they were defined by abdominal MTH (Table 5). Kohara et al. (2012) reported that the cross-sectional area of thigh muscle was negatively associated with baPWV in men, and baPWV was higher in subjects with both thigh muscle sarcopenia and visceral obesity than in those with only one abnormality [8]. This indicates that arterial stiffness is related to changes in thigh muscle sarcopenia and abdominal obesity in the middle-aged to elderly population. Previous studies have shown that the effects of exercise on arterial stiffness were observed specifically in active muscles [37–39]. Thus, SMI measured by DXA may be a better predictor than abdominal MTH for assessing arterial stiffness, given that baPWV is a whole body index of arterial stiffness.

The findings of our study are tempered by the limitations inherent to cross-sectional studies. Future prospective studies will be needed to assess the effects of SO detected by abdominal MTH on MetS and other cardiovascular diseases to obtain stronger clinical evidence.

In conclusion, this cross-sectional study demonstrated that MetS risk score was independently associated with abdominal MTH as measured by ultrasound, but not with muscle thickness in the other regions, including the thigh. HbA1c and the number of MetS risk factors in the SO group as defined by abdominal MTH were significantly higher than that in the GO group, although there was no significant difference in that between the GO and SO groups when defined by DXA-measured SMI. These findings suggest that assessing site-specific muscle loss in the abdominal region would better detect obese patients at higher MetS risk than assessing total body muscle loss. Ultrasound-derived abdominal MTH would allow a better assessment of sarcopenia in obese patients and can be used as an alternative to conventionally-used SMI measured by DXA.
